# State of art in intra-articular hip injections of different medications for osteoarthritis: a systematic review

**DOI:** 10.1186/s12891-021-04866-6

**Published:** 2021-11-29

**Authors:** Paola Emilia Ferrara, Sefora Codazza, Daniele Coraci, Giuseppe Malerba, Giorgio Ferriero, Gianpaolo Ronconi

**Affiliations:** 1 University Polyclinic Foundation Agostino Gemelli IRCCS, Rome, Italy; 2 High Intensity Neurorehabilitation, University Polyclinic Foundation Agostino Gemelli IRCSS, Rome, Italy; 3 University Polyclinic Foundation Agostino Gemelli IRCSS, Catholic University of Sacred Heart, Rome, Italy; 4 Istituti Clinici Scientifici Maugeri IRCCS, PRM Unit of Tradate Institute, Tradate, VA Italy

**Keywords:** Intra-articular injections, hip, osteoarthritis, hyaluronic acid, corticosteroids, platelet-rich plasma, pain, functional outcome

## Abstract

**Background:**

Intra-articular hip injections for osteoarthritis represent a useful instrument to reduce pain and disability in the common clinical practice. Several medications can be injected locally with different level of evidence-based efficacy.

**Objective:**

The objective of this systematic review is to investigate the effectiveness of intra-articular injections of different medications or substances for the pain treatment and the management of disability in subjects affected by hip osteoarthritis.

**Methods:**

Two reviewers selected independently randomised controlled trials published in the last 10 years, using PubMed and Scopus databases. The risk of bias was evaluated with Cochrane library assessment tool.

**Results:**

12 randomised controlled trials have been selected. We found 8 papers comparing hyaluronic acid with platelet rich plasma, with corticosteroids and with saline solution; 1 paper compares two types of hyaluronic acid with different molecular weights; 3 papers study the effects of corticosteroids alone or compared to ketorolac or saline solution.

**Conclusions:**

The studies reviewed were heterogeneous regarding sample size, level of osteoarthritis, evaluated with Kellegren-Lawrence score, medications used and follow up timings. However, we have observed that intra-articular injections of platelet-rich plasma seem to decrease pain at short term and disability at long term, in patients affected by hip osteoarthritis better than hyaluronic acid. The association of hyaluronic acid and corticosteroids could give better results compared to hyaluronic acid alone, while the use of intra-articular ketorolac and saline solution requires more studies.

## Background

Osteoarthritis (OA) may involve all joints and typically affects weight-bearing ones; the hip is the second most frequently affected after the knee [1]. Osteoarthritis is a degenerative articular disease that seriously and progressively invalidates patient’s quality of life, and represents one of the main cause of disability in over 45-year olds, with a number of about 400 million people affected worldwide [2–4].

Hip osteoarthritis has one of the highest financial burden [5], and the prevalence ranges from 6.7% to 9.2% among adults 45 years of age [6, 7], and increase to 25% in patients aged over 55 years, constituting a source of chronic joint pain and stiffness [8–10].

Intra-articular injections are increasingly considered useful to relief pain and to improve joint motion [11].

Several medications are locally injected with different level of evidence-based efficacy as hyaluronic acid (HA), platelet-rich plasma (PRP), corticosteroids (CS) drugs, non-steroidal anti-inflammatory drugs (NSAIDs).

In the last two decades, intra-articular HA injections became popular in clinical practice because the viscosupplementation (VS) appears to have mechanical and biological effects, restoring viscoelasticy of the synovial fluid, promoting shock absorption, lubrification and joint protection [12–14]. HA exerts also a notable anti-inflammatory and analgesic effects by reducing synovial inflammation sustained by proinflammatory cytokines such as interleukin-1 (IL-1), with a significant impact on pain relief and immunomodulatory effect on inflammatory cells [15]. In addition, HA has been shown to have a chondroprotective effect in experimental in vivo and in humans studies,through the stimulation of cartilage proliferation and proteoglycan synthesis, suppression of chondrocyte apoptosis, protection from oxidative damage by free radicals, degradation of catabolic enzymes and proteases and improvement of mitochondrial function [16–20].

Platelet-rich plasma is another injectable medication obtained by centrifuging once or twice an anticoagulated venous blood sample, in order to produce a plasma fraction containing a high concentration of platelets, activated by calcium gluconate, to release several growth factors stored in platelets’ alpha-granules, such as platelet derived growth factor (PDGF), transforming growth factor beta (TGF-ß), insulin-like growth factor 1 (IGF-1), epidermal growth factor (EGF), vascular endothelial growth factor (VEGF), and fibroblast growth factor (FGF) [21, 22]. These signal pathways promote bone and soft tissue joint healing [23].

The anti-inflammatory effects of intra-articular corticosteroids injections interrupt the inflammatory cascade resulting in inhibition of vasodilatation, leukocyte migration and decreasing capillary permeability. However, the alteration of gene expression and immunomodulatory effects steroid-related, are associated with inhibition of anabolic activity of chondrocytes, decreased collagen expression and additional joint damage [24].

Other intra-articular medications, such as saline solution (SS) and non-steroidal anti-inflammatory drugs, are reported in literature for hip osteoarthritis, but exiguous studies have been conducted to date about their real efficacy on pain and function; in addition very few papers compare the effectiveness of the different injectable substances in hip OA.

The objective of this systematic review is to investigate the effectiveness of intra-articular injections of different medications or substances for the pain treatment and the management of disability in subjects affected by hip osteoarthritis.

## Methods

A comprehensive literature search via PubMed and Scopus databases was conducted using the following MESH terms: *“intra-articular hip injections”* AND *“osteoarthritis”* AND *“hyaluronic acid”* OR *“steroids”* OR *“platelet-rich plasma”* OR *“saline solution”.*

The inclusion criteria were randomized controlled trials (RCTs) published in the last decade up to March 2021, in English language, including adults > 18 years affected by hip osteoarthritis (Kellgren-Lawrence score I to IV), treated with intra-articular hip injections of hyaluronic acid, corticosteroids, platelet-rich plasma and saline solution and evaluated with disability and pain outcome measures.

The exclusion criteria were papers analysing adults with hip osteoarthritis treated with other conservative therapies (oral medications, physical therapies, therapeutic exercises), with surgical treatments and all those articles not connected with human medicine and not dealing with the objective of the review were excluded (Table 1).

**Table 1:** Inclusion and exclusion criteria according to *PICO worksheet and search strategy*. US National Center for Dental Hygiene Research. Miller, S.A. (2001)

Criteria	Inclusion	Exclusion
**P**opulation	Subjects > 18 years with hip osteoarthritis (Kellgren-Lawrence score I-IV)	Subjects with hip osteoarthritis treated with other conservative therapies and/or surgical interventions.
**I**ntervention	Intra-articular hip injections with hyaluronic acid of different molecular weights, corticosteroids, platelet-rich plasma and saline solution	Other conservative therapies (oral medications, physical therapies, therapeutic exercises) and surgical treatments.
**O**utcome	Disability and pain.	
**C**omparison	Intra-articular hip injections with hyaluronic acid of different molecular weights, corticosteroids, platelet-rich plasma and saline solution	
**D**ate	RCTs published in the last decade up to March 2021	
**L**anguage	Only studies written in English were included	

**Fig. 1:**
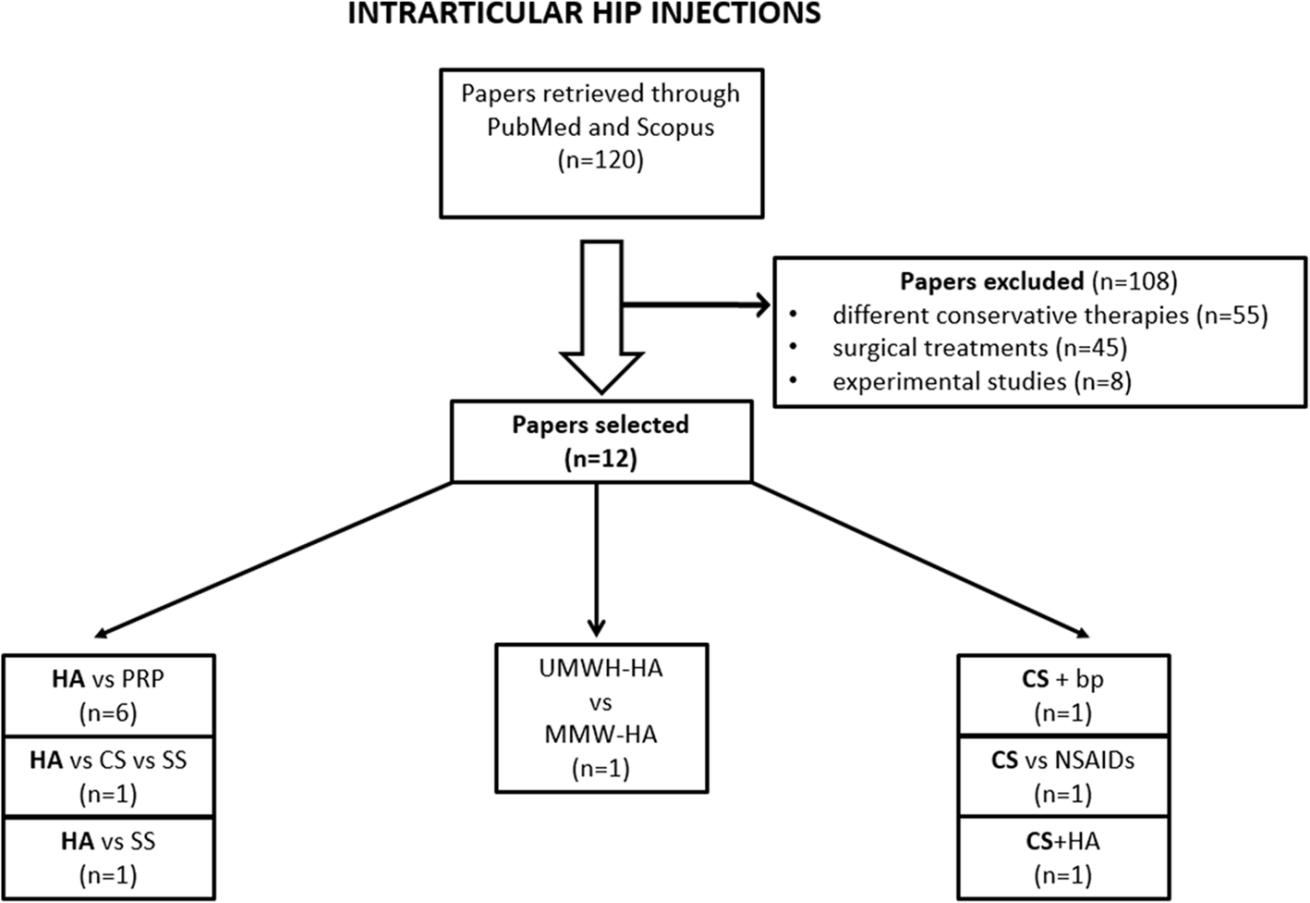
Study selection process for intra-articular hip injections. HA: hyaluronic acid; PRP: platelet-rich plasma; CS: corticosteroids; SS: saline solution; UHMW-HA: ultra-high molecular weight hyaluronic acid; MMW-HA: medium molecular weight hyaluronic acid; bp: bupivicaine; NSAIDs: non-steroidal anti-inflammatory drugs

Two reviewers (SC, PEF) selected independently the articles eligible for inclusion in the review in order to reduce the risk of inter-observer bias. Any study not approved by both of the reviewers was discarded (Fig. 1). Afterwards, the reviewers extrapolated from the articles the characteristics of the sample, the intra-articular medication injected, the trial procedures, the outcome indexes, the timing of follow-up and main results of each paper selected (Table 2).

**Table 2:** Characteristics of the studies

PAPERS	INTERVENTIONS (n. patients or hips)	TOTAL PATIENTS	K-L GRADE	INJECTIONS CHARATHERISTICS		OUTCOME		
						MEASURES	FOLLOW-UP	MAIN RESULTS
				INTRA-ARTICULAR MEDICATION	N. INJECTIONS (n/w)			
**Jurgensmeier et al. 2021** [25]	CS (30) KET (28)	58 hips	>II	Triamcinolone acetonide 80 mg **VS** Ketorolac 30 mg	1	HOOS VAS PROMIS	T0: baseline T1: 1 week T2: 1 months T3: 3 months	KET comparable improvement to CS
**Krautler et al. 2021** [26]	HA (LMW HA): (16) PRP (LP-PRP)( (20)	36 hips	II-III	Supartz® (620-1170 kDa) 2.5 ml of 1% (10 mg) **VS** LP-PRP 1-2 ml	3 1/W	WOMAC ROM	T0: baseline T1: 3 months T2: 6 months T3: 12 months T4: 24 months	Significant improvement in WOMAC and hip internal rotation for LP-PRP group at 6 months. Better WOMAC for LP-PRP until 24 months, between groups.
**De Rezende et al. 2020** [27]	CS (19) CS + 2 ml HA (19) CS + 4 ml HA (22) CS + 6 ml HA (22)	82	II-III	Hylan G-F 20® (6000 kDa) 6 ml **±** Triamcinolone acetonide 40 mg 2 ml	1	VAS ROM WOMAC Lequense	T0: baseline T1: 1 month T2: 3 months T3: 6 months T4: 12 months	Improvement in all groups for pain, function, and quality of life up to a year in HOA. CS+HA improve ROM up to one year.
**Villanova et al. 2020** [28]	HA (37) PRP (37)	74	I-IV	Synvisc-One® 60 mg/6 mL (6000 kDa) **VS** PRP 6 ml	1	VAS HHS WOMAC OARSI criteria	T0: baseline T1: 1 week T2: 1 month T3:12 months	Both groups showed improvements in VAS score at each follow-up. HA group showed a significative HHS score at T3
**Brander et al. 2019** [29]	HA (182) SS (175)	357	III	Hylan G-F 20® (6000 kDa) 48 mg/6 ml **VS** Saline solution 6ml	1	WOMAC PTGA	T0: baseline T1: 4 weeks T2: 8 weeks T3: 16 weeks T4: 26weeks	Significative improvements in both groups for all outcomes measures up to T4.
**Clementi et al. 2018** [30]	UHMW-HA (23) MMW-HA (27)	50	III	Fermathron S®, UHMW-HA 69mg/3,0 ml	1	Lequense index VAS WOMAC	T0: baseline T1: 1 month T2: 3 months T3: 6 months T4: 12months	No significant difference in the clinical outcomes between groups until T4.
				Hyalubrix® 60, MMW-HA (3200 kDa)	2			
**Doria et al. 2017** [31]	HA (40) PRP (40)	80	I-II	Hyalubrix® 15 mg/ml (3200 kDa) **VS** PRP 5 ml	3 1/W	WOMAC VAS HHS	T0: baseline T1: 6 months T2:12 months	PRP did not offer significantly better results compared with HA
**Dallari et al. 2016** [32]	PRP(44) PRP+HA (31) HA(36)	111	I-IV	PRP 5 ml **VS** Hyalubrix® HA 30 mg/2 mL (3200 kDa)	3 1/W	VAS HHS WOMAC	T0: baseline T1: 2 months T2: 6 months T3: 12 months	At all follow-ups PRP group had the lowest VAS scores, compared with HA and PRP+HA groups The WOMAC score of the PRP group was significantly better at T1 and T2, but not at T3.
**Di Sante et al. 2016** [33]	HA(22) PRP (21)	41	II-III	Hyaluronic acid 30 mg/2 ml, (1000-2900 kDa) **VS** PRP 3 ml	3 1/W	VAS WOMAC	T0: baseline T1: 4 weeks T2: 16 weeks	The functional WOMAC and VAS score in the HA were better at T2 than PRP. PRP presents significant improvement in VAS at T1
**Battaglia et al. 2013** [1]	PRP (50) HA (50)	100	II-IV	PRP 5 ml **VS** Hyalubrix®30 mg/2 ml HMW-HA (1500 kD)	3 1/ 2 W	HHS VAS	T0: baseline T1: 1 month T2: 3 months T3:12 months	PRP showed improvement in HHS and VAS as HA until T3
**Atchia et al. 2011** [34]	HA (19) SS (19) CS (19) CTL (20)	77	Croft I-IV	Durolane 3 ml/60 mg ( 90.000 KDa) **VS** SS 3 ml **VS** Methylprednisolone acetate 3 ml/120 mg	1	WOMAC NRS	T0: baseline T1: 1 week T2: 4 weeks T3:8 weeks	Significant improvement in NRS and WOMAC until T3 in CS group
**Young et al. 2011** [35]	CS 55 CS + sw: 55	110		Triamcinolone acetonide 40 mg + Bupivicaine 2 ml **VS** Triamcinolone acetonide 40 mg + Bupivicaine 2 ml + Sterile water 6 ml	1	WOMAC Oxford pain chart	T0: baseline T1: 3 months	No differences between groups

*K-L* Kellgren-Lawrence score, *W* week, *CS* corticosteroids, *KET* ketorolac, *HOOS* Hip Osteoarthritis Outcome Scores, *VAS* Visual Analogic Scale; PROMIS Global Health Scores, *HA* hyaluronic acid, *LMW-HA* low molecular weight hyaluronic acid, *LP-PRP* leukocyte-poor platelet-rich plasma, *WOMAC* Western Ontario and Mc master University osteoarthritis index, *ROM* range of motion, *PRP* platelet-rich plasma, *HHS* Harris Hip Score, *OARSI* osteoarthritis research society international, *SS* saline solution, *PTGA* Patient Global Self-Assessment, *UHMW-HA* ultra-high molecular weight hyaluronic acid, *MMW-HA* medium molecular weight hyaluronic acid, *HMW-HA* high molecular weight hyaluronic acid, *CTL* control, *NRS* numeric rating scale, *sw* steril water

## Results

The literature search identified 120 papers published in PubMed and Scopus databases as described in algorithm (Fig. 1). We excluded 108 papers: 55 studied conservative therapies for hip ostheoartritis not included in this review, 45 articles described surgical treatments and 8 were experimental studies.

Twelve RCTs were included: 8 papers about hip injections with HA compared to other medications (corticosteroids, platelet-rich plasma and saline solution), one study compared the effects of two different molecular weights HA injections; three papers analyzed intra-articular steroids hip injections, alone or in comparison with NSAIDs, hyaluronic acid and saline solution.

A total of 1176 patients were included in this systematic review: 123 were treated with CS injection, 452 with HA, 212 with PRP, 63 with CS + HA, 55 with CS+ sterile water 28 with ketorolac (KET), 194 with normal saline solution, 31 with PRP+HA; the others 18 patients represented RCTs control groups with standard care.

**Fig. 2:**
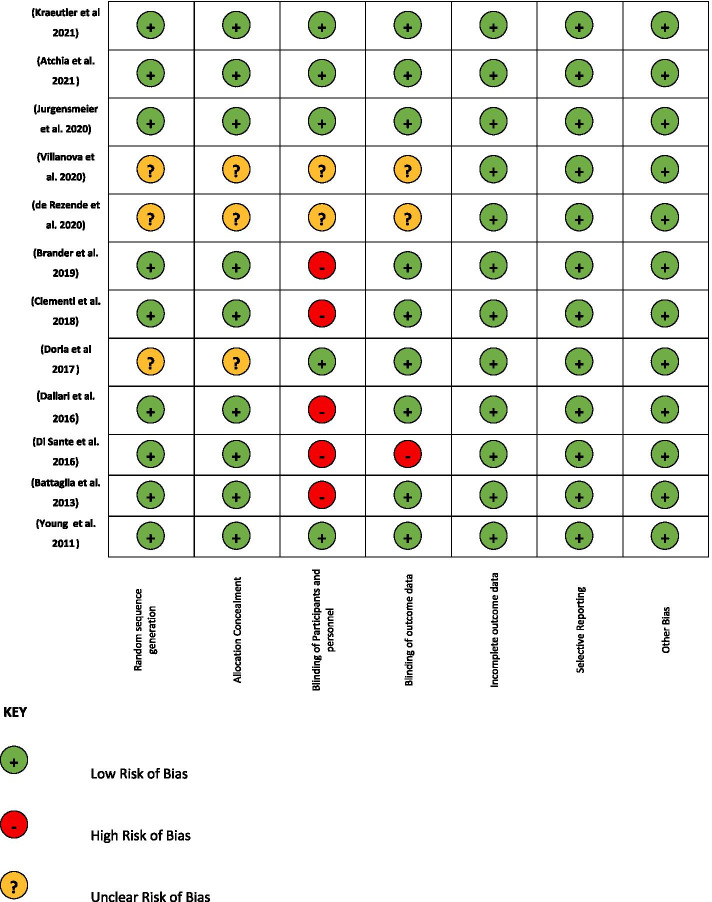
Evaluation of Bias

The Cochrane library assessment tool was used to evaluate risk of bias in the 12 RCTs according with PRISMA guidelines [36]. A green light was assigned to a low risk of bias, a yellow light to an unclear risk of bias and a red light to a high risk of bias (Fig. 2). Only 4 papers [25, 26, 34, 35] were found to have a low risk of bias (all green lights for the parameters considered). Regarding “Random sequence generation” and “Allocation concealment”, 25% of the articles were unclear, and 75% had a low risk of bias. Regarding “Blinding of participants and personnel” 41,7% had a low risk of bias, 16,6% were unclear and 41,7% had a high risk of bias.

For the evaluation of “Blinding of outcome data”, 1 paper had a high risk of bias, 24% of papers are unclear and about 75% had a low risk of bias. Considering “Incomplete outcome data”, “Selective reporting”, and “other biases”, all papers presented a low risk of bias.

## Discussion

Intra-articular hip injections have been employed for some years as a safe and effective therapeutic tool in the clinical management of painful hip osteoarthritis, with a reported complication rate of between 10 to 30% of patients [13], significantly decreased by the introduction of ultrasound and fluoroscopic guidance [37].

The objective of this systematic review is to investigate the effectiveness of intra-articular injections of different medications or substances for the pain treatment and the management of disability in subjects affected by hip osteoarthritis.

In our review we found 12 RCTs about intra-articular injections of hyaluronic acid, platelet-rich plasma, corticosteroids, saline solution and ketorolac for symptomatic hip OA, as described in Table 2.

We used the Cochrane library assessment tool to evaluate risk of bias, and it results a very low risk only for 4 papers [25, 26, 34, 35]. A high risk of bias was found for the items “Blinding of participants and personnel” for 5 papers [1, 29, 30, 32, 33] and “Blinding of outcome data” for one paper [33]. The different administrative procedure of PRP compared to HA could make difficult to realize the blinding of patients and clinicians during the treatment (Fig. 2).

All the selected papers used similar protocols for the number of injections, according with drugs data sheet.

Also outcome measures were similar in the different studies: VAS NRS, Lequense index for pain, WOMAC and HHS for functional disability, Oxford pain chart for quality of life used only in one paper [35] as described in Table 2.

The selected RCTs were heterogeneous regarding sample size, that ranged from 36 hips [26] to 357 patients, reported in the unique multicenter clinical trial [29] founded in our research.

The 1176 patients of this review presented very different levels of osteoarthritis according with Kellegren-Lawrence score [38], from I (possible narrowing of joint space medially and possible osteophytes around the femoral head; or osteophytes alone) to IV (gross loss of joint space with sclerosis and cysts, marked deformity of femoral head and acetabulum and large osteophytes), with a wide variability, also, considering Croft grade in one paper [34] from I (definite osteophytes only and measurement) to Croft IV (presence of three of the following: joint space narrowing, osteophytosis, subchondral sclerosis of > 5 mm, cyst formation) [39].

Ten RCTs, selected in this review, studied the use of hip HA injections as treatment or as control groups.

Despite the widespread use of injections with hyaluronic acid, in the literature there is no consensus about the best viscosupplementation protocol for hip OA [40, 41].

Our data showed that the molecular HA weight was different in the papers selected. Two RCTs analysed the effects of high molecular weight HA, while all the other studies [1, 27–29, 31–33] used a mean molecular weight HA, except one [26], in which a lower molecular weight HA was employed. The comparison of their clinical effects is difficult for the considerable HA heterogeneity in term of molecular weight, concentration, elasticity, viscosity of products, in patients affected by different level of hip degenerations.

However, in this review, we did not observe different improvement in pain and functional outcomes between patients treated with high molecular weight and with mean molecular weight HA until 12 months after treatment. Only one paper gave different results [30] until 12 months comparing effects of high and mean molecular weight HA.

The effects of PRP versus HA for the treatment of symptomatic hip osteoarthritis was analysed in 6 randomised controlled trials [1, 26, 28, 31–33], with different dosages of intra articular PRP, ranging from 1-2 ml to 6 ml.

PRP was found better then HA both for pain decrease and for functional improvement at short and long term [26, 32, 33]. Two authors showed, conversely, that HA had better results than PRP in pain relief until 16 weeks [33] and better functional scores at 12 months after injection [28].

We can suggest a PRP short-term efficacy for pain decrease and a HA long-term effectiveness for the improvement of function. According with literature it could be related to HA interaction with the CD44 synoviocytes receptors [42].

However there are few high quality clinical studies about the effects of intra-articular PRP hip injections [43] on pain and disability and we underline that the standard procedures for PRP production and administration protocols varies widely among studies [33, 44, 45].

The effects of corticosteroids intra-articular hip injection in hip osteoarthritis are reported only in four studies selected. Authors used two different type of steroids: methylprednisone acetate [34] and triamcinolone from 40 mg to 80 mg [25, 27, 35], which is recommended over other steroids for osteoarthritis, due to a longer lasting and more effective pain relief [46].

Our results showed that the association of HA and CS gave a better improvement for pain, function and quality of life compared to HA alone until 1 year follow-up [27]. The combined use of HA and CS may be probably more effective than HA alone in longer-lasting analgesic effects. But, to our knowledge, the RCT of De Rezende et al. 41] is the only paper of the last decade, that analysed this association. Other studies are necessary to confirm this hypothesis.

HA seemed to give less improvements than CS in pain and function until 8 weeks after treatment [34]. According to literature data [47], this review confirmed that intra-articular CS injections improve hip OA symptoms in the short- and mid-term, and the duration of pain relief is shorter compared with HA.

The study of Young et al. [35] demonstrated the effectiveness of different injected volumes (3 to 9 ml) of triamcinolone acetonide (TA) with bupivacaine and sterile water, in improving pain and function until 3 months after treatment.

Corticosteroids injections were found comparable for pain relief and functional improvement to ketorolac intra-articular injections until 3 months from the treatment [25].

Brander et al. [38] showed that saline solution could improve pain and function until 26 weeks as hyaluronic acid but additional studies are necessary to determine if the effect is due to mechanical or biological mechanism.

Despite the widespread use of intra-articular injections we founded few randomised controlled trials, published in the last 10 years. The limits of this review are the heterogeneity of papers selected regarding level of patients OA and drugs studied. It was not possible to compare the outcomes in patients’ subgroups with similar grade of hip osteoarthritis. The RCTs selected presented low sample size. The absence of blinding was a papers’ protocol bias and gave them a low quality score.

## Conclusion

Intra-articular hip injections can be a useful instrument to reduce pain and improve function in hip osteoarthrosis, however structured studies of high quality about this topic are still lacking. Although this review does not allow us to provide strong recommendations, we can observe that there is a short-term efficacy of PRP for pain decrease and a long-term effectiveness for the improvement of function in patients affected by hip osteoarthrosis. The association of hyaluronic acid and corticosteroid can give better results compared to hyaluronic acid alone, while the use of intra-articular ketorolac and saline solution require more studies.

However, more high-quality multicentric studies with higher sample size are still needed to further define evidence-based best practice for intra-articular treatment of patients with hip osteoarthrosis.

## Data Availability

Data sharing is not applicable to this article as no datasets were generated or analysed during the current study.

## Abbreviations

CS: Corticosteroids
Dex: Dexamethasone
EGF: Epidermal growth factor
FGF: Fibroblast growth factor
GAG: Glycosaminoglycan
HA: Hyaluronic acid
HHS: Hip Harris Score
KET: Ketorolac
IL-1: Interleukin-1
IGF-1: Insulin-like growth factor 1
MW: Molecular weight
NRS: Numeric Rating Scale
NSAIDs: Non-steroidal anti-inflammatory drugs
OA: Osteoarthritis
PDGF: Platelet derived growth factor
PRP: Platelet-rich plasma
Qol: Quality of life
RCT: Randomized controlled trial
ROM: Range of motion
SS: Saline solution
TA: Triamcinolone acetonide
TGF-ß: Transforming growth factor beta
VAS: Visual analogic scale
VEGF: Vascular endothelial growth factor
VS: Viscosupplementation
WOMAC: Western Ontario and Mc master University osteoarthritis index
